# A recent view about encephalomyocarditis virus circulating in compartmentalised animal population in Northern Italy

**DOI:** 10.1038/s41598-023-27828-5

**Published:** 2023-01-11

**Authors:** E. A. Foglia, G. Pezzoni, P. Bonilauri, D. Torri, S. Grazioli, E. Brocchi

**Affiliations:** IZSLER-Istituto Zooprofilattico Sperimentale Della Lombardia E Dell’Emilia Romagna “B. Ubertini”, Via A. Bianchi 9, 25124 Brescia, Italy

**Keywords:** Immunological techniques, Sequencing, Molecular evolution, Infectious diseases, Virology, Viral epidemiology, Immunology, Microbiology, Molecular biology

## Abstract

Encephalomyocarditis virus (*Picornaviridae, Cardiovirus A*) is the causative agent of the homonymous disease, which may induce myocarditis, encephalitis and reproductive disorders in various mammals, especially in swine. Despite the disease occurred endemically in pig farms since 1997, the recent increase of death experimented in Northern Italy prompted to furtherly investigate the evolution of the virus and the actual spread of the infection. Italian EMC viruses, collected between 2013 and 2019, showed an overall antigenic stability. The in-house ELISA Monoclonal Antibodies based, able to reveal changes in seven different antigenic sites, showed only sporadic and occasional mutations in considered samples and the subsequent phylogenetic analysis confirmed antigenic panel’s remarks. All the isolates could be classified within a unique lineage, which comprise other European strains and confirm that the viruses currently circulating in Italy developed from a unique common ancestor. Despite the demonstrated stability of virus, some putative newly emerged variants were detected through antigenic profile analysis and phylogenesis. Finally, the serosurvey proved that spread of EMCV is greater than the diffusion of fatal infections would suggest, due to subclinical circulation of EMCV. It demonstrated an increase in the proportion of seropositive farms, if compared with previous data with no remarkable differences between farms with and without clinical evidence of disease.

## Introduction

Encephalomyocarditis virus (EMCV) is a single-stranded RNA virus belonging to *Picornaviridae* family, genus *Cardiovirus,* which acts as causative agent of the homonymous disease*.* EMCV was first isolated in 1940^1^ and could infect many different mammals, including domestic, captive and wild animals. Swine is the most receptive between livestock species^2–6^ and for this reason, the virus often circulates endemically in areas characterized by intensive pig farming^7^. In infected hosts, EMCV may cause acute and lethal myocarditis and/or encephalitis, with death rate usually depending to aging. It approach 100% in offspring of suckling or pre-weaning piglets^8^; while in post-weaning to adult animals, infection is usually subclinical and mortality depends on the onset of other complications^9^. Interestingly, in pregnant sows EMCV may induce reproductive disorders, such as abortion or mummified foetuses and stillbirths, suggesting viral vertical transmission^9–12^.

In certain countries, EMC infection shows a typical seasonal tendency, with peaks during autumn/winter season^7^. This trend seems to originate from the habits of rodents population, especially rats, to shelter in farms and zoos during the cold season. It is proven that rodents work as natural EMCV reservoir and nestling in stables would transmit virus to livestock animals and captive animals of zoos, almost surely following a faecal-oral path of transmission^3,13–15^. In addition, some species of rodents show a peculiar specificity of EMCV to affect pancreas, inducing diabetes mellitus. This singularity has never been detected in any other mammal^16–18^.

In human population both clinical and subclinical circulation of virus were detected, suggesting that EMCV has also a dangerous zoonotic potential for humans. Indeed, the virus was isolated from two febrile human patients in Peru in 2004, despite myocarditis in the described clinical cases was not as severe as that observed in swine^19–21^. Furthermore serological surveys of human population proved a good part of tested people is EMCV seropositive, demonstrating that in human the virus is circulating widely than the expected, especially in developing countries, with low level of sanitation and high probability of faecal-oral transmission^21–23^.

The infection, detected in USA since early ’40, nowadays is widespread in many countries, both industrialized and not industrialized^5,7,19,24–27^. In Northern Italy EMCV infection was first described in 1986 and, after sporadic detections in the following ten years, from 1997 the disease occurred endemically in pig farms, causing almost exclusively fatal myocarditis in adult animals^2,4,7,13,28,29^. The resulting economic losses, depending on the seriousness of symptoms and consequent mortality rate, had been critical for farmers. As follow up of two previous EU funded research projects conducted during 1996–2002, the present study aims to give more useful information about EMCV diffusion in Northern Italy, by means a phylogenetic analysis and evaluation of the antigenic profile of isolates collected between 2013 and 2019. In addition to the characterization of circulating strains, a serological survey of pig farms in study area in 2016 will evaluate the real extent of viral spread, highlighting its subclinical circulation. Comparison with previous analogous analyses may provide also data about changes in viral diffusion over time.

## Materials and methods

### Origin of viruses

**Figure 1:**
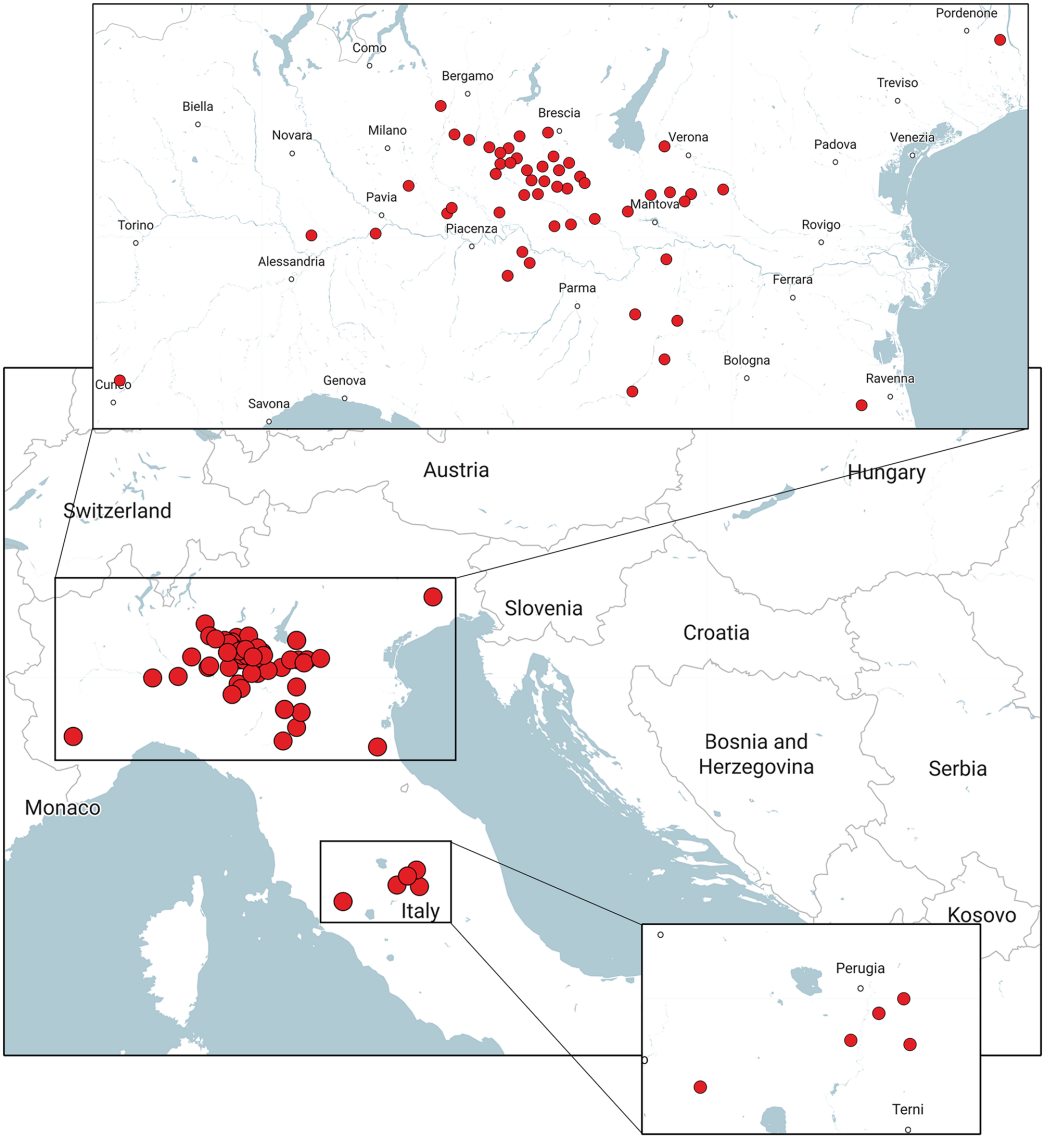
Geographic origin of EMCV recent isolates. The map shows the exact location of samples’ collection. The main box is an overall picture of the sampling area involving Northern and Central Italy; two boxes display magnifications of affected area. The major study area, big square, included almost all of the Po Valley, primarily Lombardy, Emilia-Romagna and Veneto, even if analysis also considered some samples from Piedmont (Cuneo, in the south-western border of the box) and Friuli Venezia-Giulia (near to Pordenone, in the north-eastern border of the box). Affected Central Italy area, tiny square, was smaller and included a zone between south of Tuscany (close to Grosseto) and Umbria (province of Perugia). Each red dot represents one village where sampling took place. Two or more farms could be located in the same village and indicated by a unique icon. Pictures were generated on-line on https://it.batchgeo.com/.

Most of samples considered in this research (66 out of 72) were collected from swine farms with cases of fatal myocarditis. All sampling sites were located in an endemic region with a history of clinic EMC comprising Northern and Central Italy (Fig. 1). Each outbreak herd was usually considered once, so, most of the analysed organ homogenates of pig hearts originated from different farms. Sequential sampling of a single farm occurred rarely, in conjunction with a reappearance of the disease (samples and isolates’ information are summarized in Supplementary Table S1). A small number of considered samples, formerly six, originated from different species, one porcupine and one macaque housed in a WWF Rescue Centre for Wild and Exotic Animals in province of Grosseto, Tuscany^4^ and four lemurs deceased in subsequent years in the natural park “Parco Natura Viva” in province of Verona, Veneto. All samples were collected between 2013 and 2019.

### Viral isolation

**Figure 2:**
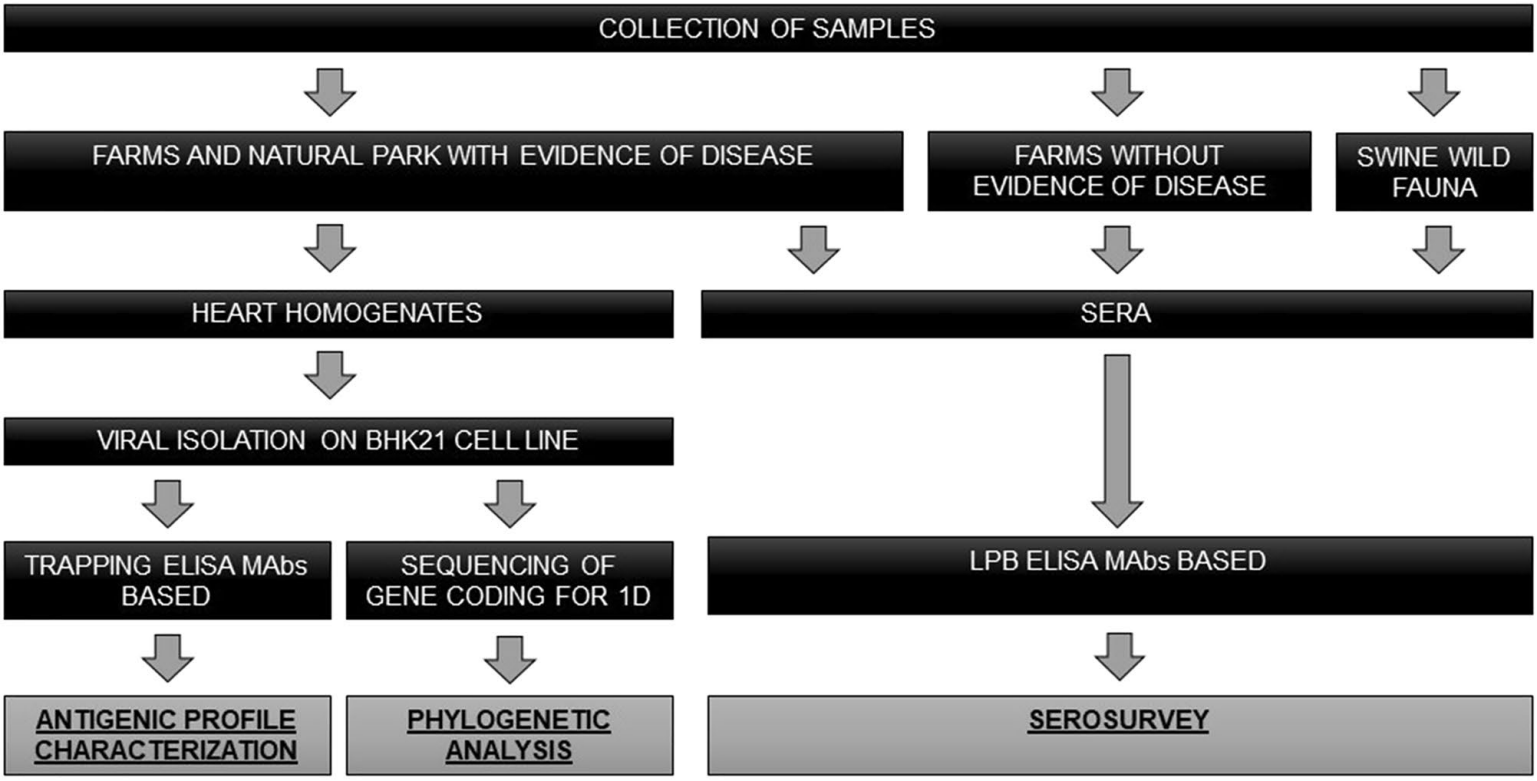
Workflow. The diagram shows the pattern of activities and experiments carried out, from sampling to obtainment of data. In dark cells, all the operations performed to get the results, presented in lighter cells.

The 72 selected heart homogenates, all positive to the diagnostic conventional RT-PCR (data not shown), were inoculated on monolayer of BHK-21 cell line for isolation, after sterilization by filtration with 0.45 μm filters. Further passages (maximum three) were conducted until complete CPE, as previously described^9^. Later, isolates were stored at −80 °C. Workflow diagram summarize treatments and analyses of samples (Fig. 2).

### Trapping ELISA monoclonal antibodies (MAbs) based

A panel of 39 MAbs, composed by 19 non-neutralizing and 20 neutralizing elicited against the first Italian EMCV strain ITL-135/86 detected in 1986^30–34^, was used to characterize the antigenic variations among the EMCV isolates, by means of a trapping ELISA.

Briefly, ELISA microplates were coated with a saturating concentration of anti-EMCV rabbit serum and viruses, as supernatant of infected BHK-21 cells, were trapped. Each MAb (at a previously titrated dilution) was then incubated, followed by an anti-mouse IgG HRP-labelled antibody. Each step required an incubation at room temperature for 1 h.

The reactivity of each MAb with field isolates was expressed as a percentage of the corresponding reaction with the homologous strain, assumed to be 100%.

### RNA extraction and RT-PCR

Viral RNA was extracted from isolates (Fig. 2) using QIAamp Viral RNA Mini Kit (QIAGEN, Hilden, Germany).

Reverse transcription and polymerase chain reaction were conducted consequently in a single step reaction, using QIAGEN One-Step RT-PCR Kit (QIAGEN, Hilden, Germany).

Two specifically designed primers (Metabion International AG, Planegg/Steinkirchen, Germany), formerly EMCV-FF2 and EMCV-REV1 (Table 1B), were used to amplify a 1055 nucleotide region, which includes the whole coding sequence for capsidic protein VP1 (gene 1D). RT-PCR assay was performed in a 25 μL reaction mixture containing 5 μL extracted RNA, 5 μL 5X Reaction Buffer, 1 μL dNTPs-mix, 1 μL Enzyme Mix, 600 nM each of forward and reverse primers and 5U RNasin® Ribonuclease Inhibitors (Promega, Madison, WI, USA). The applied thermal cycling profile is briefly summarized in Table 1A.

**Table 1:** Specifics of RT-PCR VP1 targeted.

A				
Step	Temperature	Time	No. cycles	
Reverse Transcription	50 °C	30'	1	
Reverse-transcriptase inactivation/Taq Polymerase activation	95 °C	15'	1	
Denaturation	95 °C	30''	40	
Annealing	53 °C	30''		
Elongation	72 °C	1'		
Final elongation	72 °C	7'	1	
–	4 °C	∞	–	

B				
Primer name	Sequence 5'-3'		Genome region targeted	Use
EMCV-FF2	ACCTCAGCCAAGATCCTTACA		1C (VP3)	RT-PCR-Seq
EMCV-REV1	CTCAGAATCACGTCCGCA		2A	RT-PCR-Seq
EMCV-FFint3	GGCTTTGCACCTTTCTCCAA		1D (VP1)	Seq
EMCV-REVint3	GCTTCGTCCCTGCAAAGAAA		1D (VP1)	Seq

Tables show technical specifics of RT-PCR reaction used for amplification of 1D gene (coding for VP1) of EMC virus. A, thermal profile of RT-PCR, including temperature, duration and repeats of each step. B, primer used for amplification of 1D and for Sanger sequencing.

### Nucleotide sequencing and phylogenetic analysis

PCR products were run on a 2% agarose gel and those of the expected size were purified using NucleoSpin Gel and PCR Clean-up kit (Macherey–Nagel GmbH & Co. KG, Düren, Germany). Purified amplicons were sequenced with Applied Biosystems 3500 XL Genetic Analyzer by an automated fluorescence-based technique following the manufacturer's instructions (Thermo Fisher Scientific, Waltham, MA, USA). In addition to those used for amplification, two supplementary internal specific primers were used for sequencing (Table 1B). Obtained fragments were assembled in a unique sequence for each strain using SeqMan Pro-Lasergene 10 Core Suit software (DNASTAR, Madison, WI, USA). Phylogenetic analysis was carried out with MEGA-X software^35^ by Maximum Likelihood (ML) method^36^ with Tamura-Nei (G + I) model^37^. The analysis included also EMCV 1D sequences available online (https://www.ncbi.nlm.nih.gov/nuccore; Supplementary Table S2) in addition to those obtained from sequenced isolates. The confidence of the obtained tree was assessed by bootstrap analysis with 1000 replicates^38^.

### Sera collection

Porcine sera tested for serological positivity against EMCV were selected from those received under SVDV Eradication Plan by National Reference Laboratory for Vesicular Disease in IZSLER, Brescia (Italy). The number of sera collected per farm may change, depending from the size of the farm, the required minimum detectable percentage of seroprevalence and the demanded confidence level. This study considered exclusively sera sampled from farms in order to detect seroprevalence ≥ 10% with a confidence level of 95%. The farms interested were comparable in size and involved both those with and without evidence of viral circulation (Fig. 2). Overall, 415 farms were involved in 2016 survey, for a total of 15,996 swine sera. That investigation was compared to analogous serosurvey carried out in 2000^29^ and 2010, which involved respectively 5,696 and 16,483 sera. Results from the survey were divided in positive and negative, and sub-grouped by the features of the farm of origin (Fattening or Breeding). Differences between groups’ results were tested with a one-sided Fisher’s exact test, in order to assess its statistic reliability. Serosurvey included also some sera obtained from sampling of wild boar population (Table 2) of mountain regions of Alps (Lombardy) and Apennines (Emilia-Romagna).

**Table 2:** Serosurvey of wild animals from mountain regions.

			Sample size
**ALPS**			
POS	3	8%	38 sera
NEG	35	92%	
**APENNINES**			
POS	11	5%	204 sera
NEG	193	95%	

The table shows origin and number of tested sera and seroprevalence of swine feral population.

### Liquid phase blocking ELISA (LPBE) for serological monitoring

Swine sera were tested to check the presence of Abs against EMCV using a MAbs-based LPBE developed, validated and routinely used in IZSLER, Brescia (Italy). Briefly, inactivated EMCV antigens (ITL-135/86) were incubated with porcine sample sera, moved to a microplate coated with a polyclonal swine serum and then a specific HRP-labelled MAb was added. Each step requires an incubation at room temperature for 1 h. The ability of a serum to inhibit the binding of the antigen to the adsorbed MAb was evaluated through colorimetric reaction^33^. Each single sample was considered positive if its competition percentages was ≥ 80%. Depending on the percentage of seropositivity the farms were divided in:

- Postive Farms, with seroprevalence > 10%
- Negative Farms, in turn divided in :
  o Farms with null seroprevalence
  p Farms with seroprevalence ≤ 10%

The obtained seroprevalence data and differences between them were statistically validated with one-sided Fisher’s exact test and *p* value obtained for every couples compared was lower than 0.001.

### Ethical statement

Ethical review and approval were waived for this study that did not involve killing of animals. The samples did not originate from experimental trial but took advantage just from diagnostic activity of National Reference Laboratory for Vesicular Disease in IZSLER, Brescia (Italy) under the National SVDV Eradication Plan. Therefore, since the sampling was not specifically programmed as experimental study, but originating from diagnostic activity, we believe that it does not fall in the provisions of the National Law (e.g. DLSG 4/3 2014, n. 26. Application at national level of the EU Directive 2010/63/UE) and no ethical approval or permit for animal experimentation was required. Sera collection, planned under an Italian Institutional Activity, did not needs of informed consents provided by the original owners of the animals, and neither consents for future research efforts were solicited to the owners of the herds. EMCV serosurveillance returned only aggregated data and any possibility of tracing back from a single pool to the farm of origin is made impossible, in order to protect owners’ privacy. Only the scientific manager of the project had the ability to match sera with some information about the farms (i.e. location, type of herd, size, etc.), but never name of the farm or of the owners, according to anti-trust policy of the Institute.

## Results

### Virus isolation

Only 68 samples, out of 72, successfully infected BHK-21 cell line, replicating and inducing complete cytopathic effect. Virus isolation of the remaining five samples resulted negative after the third passage.

### Characterization of circulating strains

#### Antigenic profile analysis

**Figure 3:**
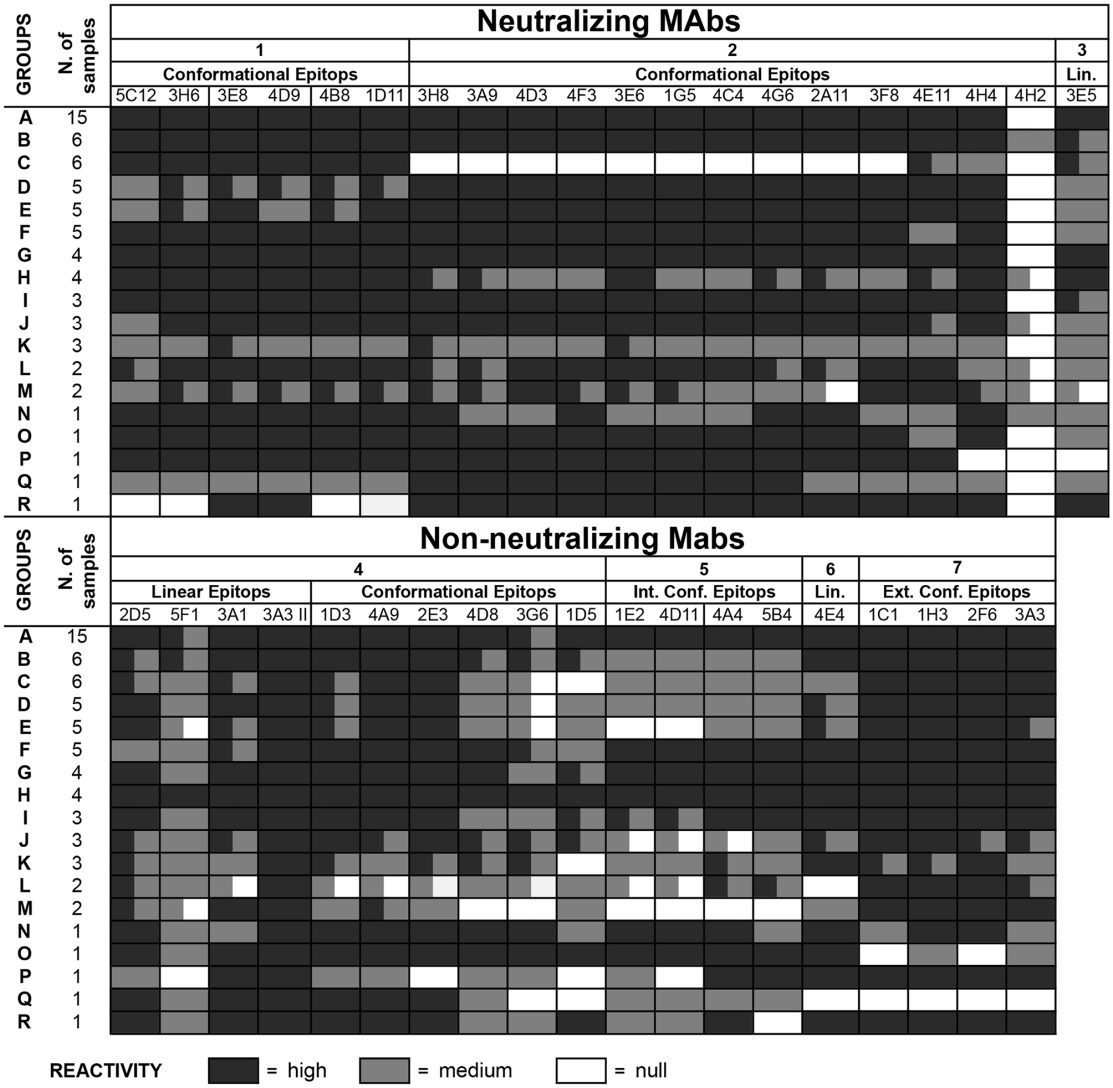
Characterization of antigenic profile of recent isolates. Graphical representation of reactivity of MAbs against recent EMCV isolates considered in the analysis. The upper rows of the tables indicate MAbs features: names, epitopes’ characteristics, areas and eventual involvement in viral neutralization. Letters from A to R, on the left, indicate the groups generated clustering analysed samples accordingly to homogeneity in induce antibody reactivity, and the size of each group is indicated alongside. Colours of squares denote reactivity level: black, high; grey medium; white, null. The picture revealed an overall homogeneity of reactivity of MAbs listed in area 1, 2 and 7 and a discrete, but occasional and unfixed, variability of the other MAbs. Maintenance of high reactivity of 3A3 II (completely black column) and loss of reactivity of 4H2 (almost completely white column) are evident in the picture. Even changes in epitopes of area 2 of samples clustered in Group C, with consequent loss of reactivity by specific MAbs, are clearly noticeable.

The 68 isolates (Supplementary Table S1) were tested using the panel of 39 MAbs, able to identify seven antigenic areas, which include multiple epitopes, conformational and linear epitopes, internal and external epitopes. 20 MAbs (Fig. 3) are also involved in virus neutralization^33,34^.

Investigation through trapping ELISA test and elaboration of results (as described in 2.3) provided a percentage of reactivity with respect to the homologous strain. In order to obtain simpler and more understandable data, those percentages were classified in three different levels: high, when it was higher than 70%; medium, when it was between 70 and 25%; and null, when it was lower than 25%. After this classification, isolates were clustered according to homogeneity in reactivity, in 18 groups. Each group was composed by one or more isolates, and listed from A to R from most to least numerous.

The obtained profile revealed an overall antigenic stability of considered samples, particularly strong in area 1, 2 and 7 (Fig. 3). The first two areas are characterized by conformational epitopes recognized by neutralizing MAbs both in intact and partially degraded capsids^34^. The antibodies able to identify epitopes included in these areas maintained high reactivity against 75% of tested samples, except for MAb 4H2. Area 7 is also made of conformational epitopes, but they are recognized by non-neutralizing MAbs and only in whole capsids^34^. In that case, 95% of samples were identified with very high reactivity level (Fig. 3).

Another highly conserved antigenic site was a linear epitope of VP3; MAb 3A3 II (area 4), which identifies that epitope, fully reacted against all tested samples (Fig. 3).

Remaining antigenic areas, all detected by non-neutralizing MAbs (areas 4, 5 and 6) showed a discrete variability. The most variable epitopes lay in area 5, and specific MAbs against this region showed unaltered reactivity levels only against 45% of samples (Fig. 3).

Two antigenic sites, respectively recognized by MAbs 3G6 and 4H2, showed a very significant antigenic variability. Reduction in reactivity of the first antibody, a non-neutralizing MAb recognizing conformational epitopes of area 4, was milder and interested only a small part of viral population; its reaction was weaker against 37% of samples and completely lost against 25% (Fig. 3). The neutralizing MAb 4H2, involved in detection of a conformational epitope in area 2, revealed more severe changes. The antibody detected only 4% of selected isolates while in 80% of cases its reaction completely failed; the remaining 16% corresponded to milder reactivity (Fig. 3).

**Figure 4:**
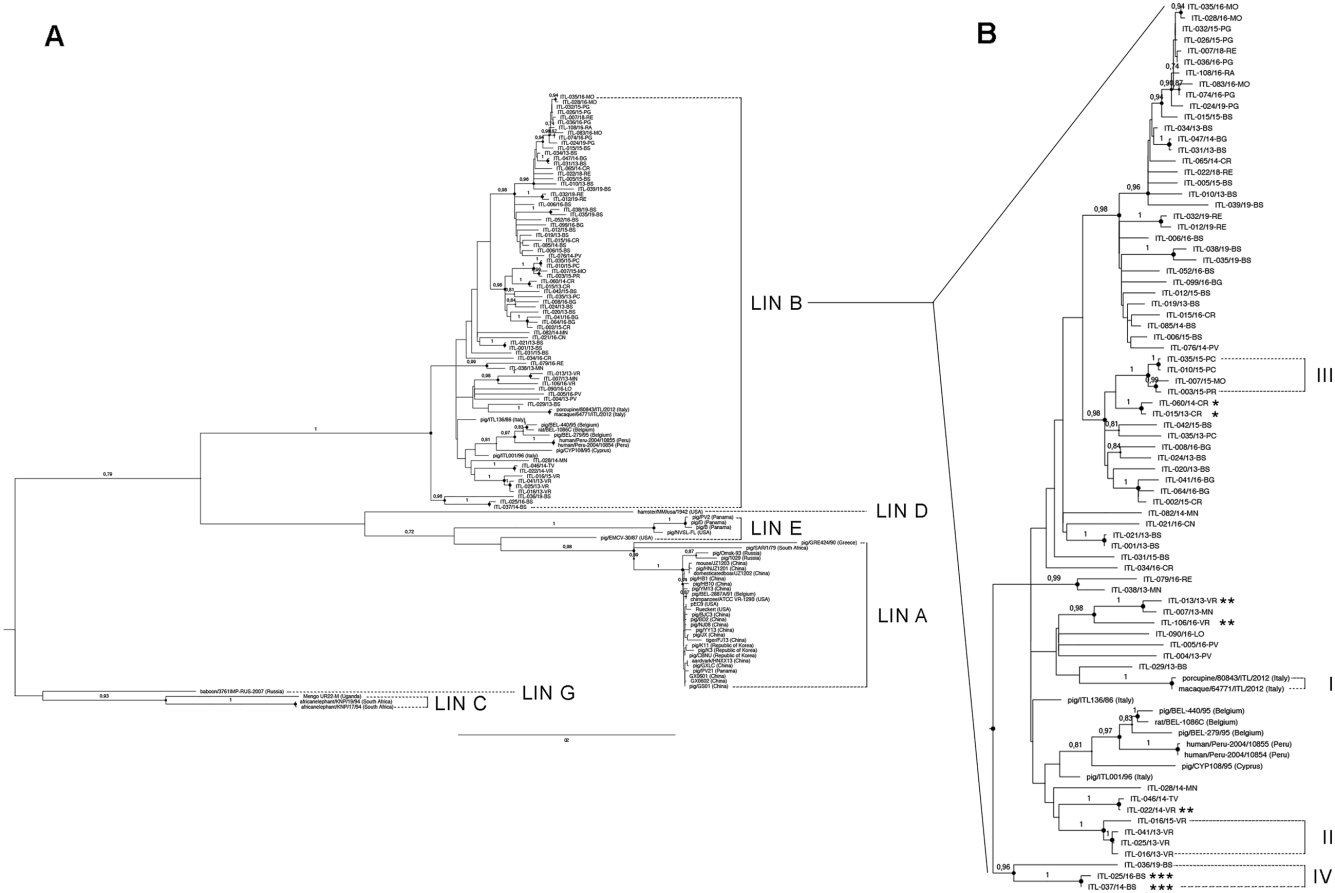
Maximum Likelihood phylogenetic tree of EMCV strains. Phylogenetic analysis of EMCV strains based on sequences of gene coding for 1D (VP1) capsidic protein. (**A**) analysis included recent EMCV isolates and strains collected in databases, respectively listed in Supplementary Table S1 and S2. Resulting tree is obtained by Maximum Likelihood method with 1000 bootstrap (see text). Sequences, all belonging to EMCV-1, clustered into 6 linages (LIN A – LIN G, sequences of LIN F were not included in the analysis), accordingly to Vyshemirskii and colleagues^40^. (**B**) magnification of LIN B, including all the samples considered by the analysis. Brackets indicate closer subgroups: (I) strains isolated from wild hosts in the recovery centre for wild animals near to Grosseto (Tuscany); (II) strains isolated from primates in the natural park “Natura Viva” in province of Verona (Veneto); (III) strains isolated in 2015 in Emilia-Romagna and clustered in Group C of Antigenic Panel; (IV) strains isolated from samples originated from an unique village in province of Brescia (Lombardy), showing higher phylogenetic distance. Asterisks sign strains originated from consecutive samplings in three farms; * farm located in Province of Cremona (Lombardy) with samples very closely clustering; ** farm located in province of Verona (Veneto) and distributed in various groups; *** farm located in province of Brescia (Lombardy) with evidence of strains clustering together. Bootstrap values are indicated over the branches, when lower of 0.7 they are omitted.

Finally, all MAbs of Area 2 except for 4E11 and 4H4, completely lost their reactivity against isolates of group C. Interestingly, four of those strains clustered very closely in the phylogenetic tree (Fig. 4B; III).

#### Molecular characterization

Phylogenetic analysis of isolates’ sequences coding for VP1 (1D), combined with those available on-line, provided data to carry out the molecular characterization of EMCV strains circulating in Northern Italy (Supplementary Tables S1 e S2).

The obtained ML phylogenetic tree (Fig. 4A) confirmed the possibility of grouping all the analysed strains into six out of seven lineages of EMCV type 1, as previously proposed^39–42^. The databased sequence belonging to LIN F was not included in the analysis (Fig. 4A).

All sequenced isolates laid in LIN B, with other European strains (Belgian and Cypriot) and closely related to the older Italian strains previously analysed (ITL-138/86 and ITL-001/96; Fig. 4A), with a percentage of divergence between 3.38% and 9.76% (mean 7.02%). Intriguingly, also the unique EMCV strains isolated from human hosts^19,20^ clustered in the same lineage with EU strains, despite coming from Peru. LIN B lays apart from that including an historical and typical strain of EMCV, the Mengo-M strain, whose 1D sequence differs about 21% from those of the strains included in the other Lineages.

A depth analysis of LIN B emphasized that samples from “Natura Viva” Natural Park and from the WWF Rescue Centre grouped in two smaller clusters (both with bootstraps equal to 1 and divergence of 1D sequences lower than 0.6%), highlighting higher phylogenetic proximity between viruses circulating in those specific, limited and closed spaces (Fig. 4B; I and II).

As mentioned in section "Origin of viruses", three farms with frequent EMC cases situated in endemic area were selected to monitor infection over time, focusing on phylogenetic proximity of viral strains circulating in one single farm in different years. In two herds, isolates collected from the same farm in subsequent years clustered closely together in specific and small groups with origin nodes of appreciable bootstraps (1 and 0.98 respectively; Fig. 4B; * and ***). Contrariwise, viral strains collected contemporarily or subsequently from the third farm were distributed in different clades in the phylogenetic tree with an appreciable genetic variability (Fig. 4B; **).

Distribution of the remaining Italian isolates in the lineage did not show clusters with geographic or temporal peculiarity (Fig. 4B). Only three samples, collected from farms located in the same village in Northern Italy, in province of Brescia, did not follow that trend and placed apart in respect of all the other European isolates, but still clustering in LIN B, (Fig. 4B; IV).

### Serological monitoring of farm and wild animals

The serosurvey of swine population of the area comprising Lombardy and Emilia-Romagna was carried out to define the real extension of EMCV diffusion in Northern Italy and the frequency of viral sub-clinical circulation.

**Figure 5:**
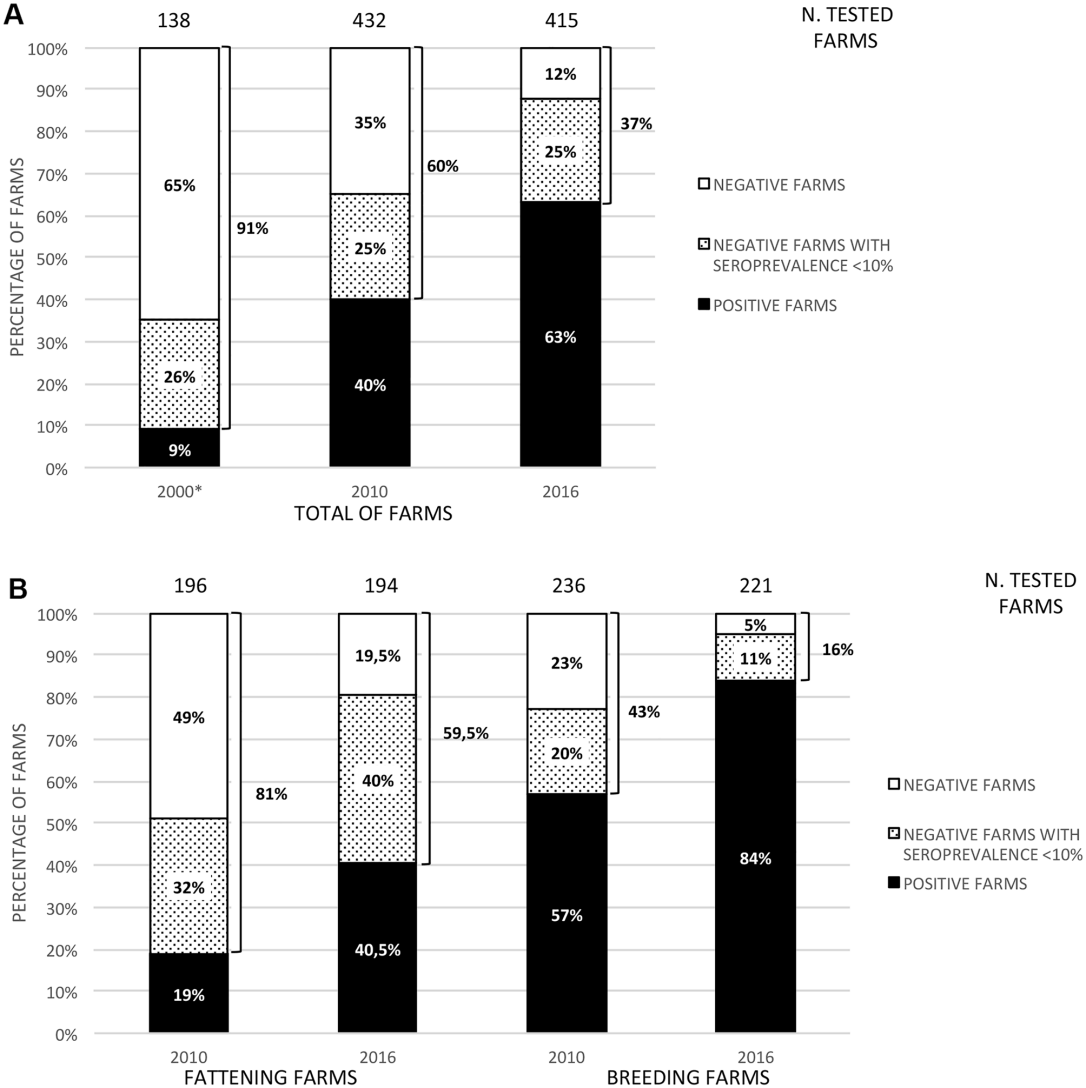
Serosurvey of pig herds in Northern Italy. Graphs compare seroprevalence percentage obtained during serosurvey of swine farms of Lombardy and Emilia-Romagna in 2016 with data of 2010 and 2000. Farms were considered as positive (black) if percentage of seropositive sera was higher than 10% and negative with lower percentage of seropositivity. Negatives include farms with seroprevalence equal to 0 (white) and with seroprevalence lower than 10% (dotted). (**A**) total of farms considered.(**B**) herds divided between Fattening and Breeding. Only 2016 and 2010 data considered. Differences between data were statistically validated with one-sided Fisher’s exact test and *p* value obtained for every couples compared was lower than 0.001. *, in 2000^29^, serosurvey divided farms by seroprevalence percentage in four groups: neg, < 5%, 5–15%, > 15%. All farms with seroprevalence lower than 15% were considered as “negative with seroprevalence” (dotted).

Obtained data revealed that the percentage of positive farms in 2016 was 63% and comparing that data with the corresponding of previous years a statistically significant increase (*p* value < 0.001) in the number of positive farms was evident (Fig. 5A). Positive farms were 40% in 2010 and only 9% in 2000^29^. Interestingly, the rate of negative farms with seroprevalence lower than 10% remained unchanged over time (26% in 2000^29^, 25% in 2010 and 26% in 2016) revealing that changes affected only positive and null farms (Fig. 5A).

Dividing the farms into two groups, between Fattening and Breeding, a huge difference was also observed. Even if, in both groups, an increase of seroprevalence from 2010 to 2016 was noticed, the percentage of positive herds was greater in breeding ones (Fig. 5B). In fattening seroprevalence growth from 19% to 40.5%, while in breeding farms from 57 to 84% in 6 years (*p* value < 0.001; Fig. 5B).

On the other hand, to split farms into two groups, the first including those with diagnosed EMC cases and the second containing those free of clinical disease, did not highlight considerable differences in percentage of seropositive herds (data not shown).

Interestingly, serosurvey of boars’ population revealed a very low circulation of virus in wildlife, with only about 6% of animals seropositive against EMCV (Table 2).

## Discussion

Viral encephalomyocarditis is a widespread pathology, which may interest both livestock and wildlife species^3,7,43^. Despite serological data demonstrated that EMCV circulate widely in pig and human populations^23,28,44^, mortality is often limited to piglets and new-borns^10,12^. Due to these concerns, viral EMC is quite underestimated and surveillance, controls and analyses of viral circulation and infection incidence are not always routinely practiced. Even if economic losses in farms depends on severity of clinical signs and mortality rates, the systematic deceases of whole litters of pigs or of pregnant sows during cold season could have a serious economic burden. Moreover, capability of EMCV to infect also human, demonstrated both by diagnosed viral EMC cases and by seropositivity of population^19–23^, make imperative to monitor virus evolution, in order to avoid the arise of new zoonotic menaces.

The aims of this work were to gain an overall idea, as detailed as possible, of current situation in Northern and Central Italy, in respect of EMCV diffusion and focusing on the characterization of circulating strains.

The analysis of interaction between viruses and specific MAbs provided information on variability of protein structure of exposed antigens. Antigenic profile of considered samples revealed an overall antigenic stability (Fig. 3), which indicates that only minor evolutionary changes of capsid structures took place over the years in EMCV strains circulating in Italy. Changes affected some areas more than others do. Data suggested the most conserved were the externally exposed epitopes, probably those inducing immune response in hosts and involved in neutralization (Fig. 3). Some minor and occasional antigenic variations were detected, mainly charged to areas not involved in neutralization (Fig. 3). All those changes were not geographically or temporally related and should represent a usual, ordinary and casual variability of viral population in a time span of few years. Even, the very evident antigenic change, which caused the complete lack of reactivity of MAb 4H2 and which comprised almost all tested isolates (Fig. 3), could be associated to a mutation fixed in the population since 1988^29^, corroborating the idea of a high stable circulating population of EMC virus in Italy.

The overall antigenic stability was also confirmed by phylogenetic analysis (Fig. 4).

Phylogenetic investigation was based on 1D gene, according to the assumption that VP1 might be subjected to selective pressure and variability. In Picornaviruses, VP1 is an external exposed capsid protein and is involved in viral pathogenicity, receptor binding and immune modulation^45–47^. Therefore comparing sequences of this region obtained from different isolates might give exhaustive information about evolution of EMCV in the considered period.

Indeed, performed investigation displayed that Italian samples were closely related with older European EMCV strains from Italy, Belgium and Cyprus, confirming the presence of a lineage (LIN B) including almost all European strains^29,39–42^. Circulating (and sequenced) strains demonstrated a very close relationship, proving their evolution from a unique ancestor (Fig. 4A).

Nevertheless, this stability present two interesting exceptions. Antigenic analysis revealed that a sub-population, called Group C (including four isolates collected in 2015 from farms located in a restricted area of Emilia Romagna and two other isolates respectively from 2014 and 2019 in neighbouring areas) presented a peculiar antigenic profile. Ten out of twelve specific MAbs elicited to react against almost three conformational epitopes^29^, did not recognize those isolates (Fig. 3). Interestingly, phylogenetic tree revealed that the four isolates form Group C collected in 2015 belonged to a specific clade, originated by a common ancestor with bootstrap value equal to 1 (Fig. 4B; III). It suggests that a new mutation took place before 2015 in a specific area, which fixed in a restricted sub-population and provided new antigenic features to the involved strains.

Phylogenetic tree disclosed the presence of another cluster, with bootstrap value of origin node 0.96, comprising three isolates sampled in 2014, 2016 and 2019 in two distinct farms in the same village in Lombardy (Fig. 4B; IV). It was included in the same lineage (LIN B) of all the other Italian strains, with a percentage of similarity higher than 85.8% (data not shown), but it seemed to develop independently from the other strains even though originated from the common ancestor of LIN B.

Unfortunately, samples from Brescia (BS) 2019 were not infective and it was not possible to isolate them on BHK-21 cell line; the consequent absence of an antigen profile does not corroborate molecular analysis of that cluster.

Characterization of the recent EMCV strains circulating in Italy demonstrated their high stability, both antigenic and molecular, and indicated that no new strains belonging to other lineages was introduced in recent years. Despite these characteristics, analysis highlighted that the onset of mutations, its fixation in the population and the emergence of strains with new putative features can potentially occur also in a stable virus, such as EMCV.

Serological monitoring of swine population corroborated previous data about diffusion of virus in the farms of Lombardy and Emilia-Romagna. The current percentage of seropositive herds (farms with seropositivity > 10% with confidence of 95%) compared to those observed in 2010 and 2000^29^ demonstrated that in the last two decades Northern Italy has been involved in a significant increase in virus exposure (Fig. 5). These data, together with a lack of increasing of fatal cases (data not shown), proved once again that EMCV circulate sub-clinically and that Italian swine population is continuously in contact with the virus, which almost certainly increased its circulation within compartmentalised swine population. Interestingly, serosurvey also revealed that Breeding and Fattening herds showed different levels of seroprevalence. Low seroprevalence of Fattening farms may be due to faster animals’ turnover; perhaps, the entrance of an EMCV positive animals, even if infected, do not cause viral spread, confirming the low incidence of pig-to-pig transmission^43^. Conversely, in Breeding farms, where higher levels of seroprevalence were detected, the long stay of animals in a unique or in very close stables, repeatedly exposes them to seasonal contact with the same reservoir population, highlighting on key role of rodents in viral circulation. Also the low seroprevalence of wild boars (Fig. 5) may confirms this outcome about rodents, revealing that isolates animals, which are less related to rats or other infected rodents, have less occasion to encounter EMC virus.

Even the close phylogenetic relationship, often detected between the strains affecting farms, Natural Parks and rescue centres over time (Fig. 4B; I, II, IV, * and ***), strongly suggested the importance of the role played by the reservoir populations in the consecutive and continuous introduction of virus in these closed spaces.

Concluding, this better knowledge of viral EMC, an often-neglected disease with potentially high socio-economic repercussions, would be crucial to contain viral spread and to reduce economic losses in endemic areas, shifting the focus on the rodent populations infesting herds and their surroundings.

Moreover, the zoonotic potential of strains closely related to Italian variants combined with the demonstrated sub-clinical circulation in livestock animals continuously interacting with humans make EMCV a special guard. Timely updates about its molecular and antigenic changes would be essential to keep every possible complication under control.

## Supplementary Information

Supplementary Information 1.

Supplementary Information 2.

## Data Availability

The datasets generated during and/or analysed during the current study are available in the NCBI/nucleotide repository, with the following Accession Numbers: OL840475-OL840544; OL963559; OL963560.
