# Supplementary material for: A recent view about encephalomyocarditis virus circulating in compartmentalised animal population in Northern Italy
Source: Sci Rep. 2023 Jan 11;13:592. doi: 10.1038/s41598-023-27828-5 (PMC9834260; doi:10.1038/s41598-023-27828-5)
Supplement: Supplementary file 1 — Supplementary Information 1. [file 41598_2023_27828_MOESM1_ESM.docx]

|  |  |  |  |  |  |
| --- | --- | --- | --- | --- | --- |
| **Isolates designation** | **Gene Acc. No.** | **Geographic origin (Italian Province)** | **Year** | **Species** | **Analysis** |
|  |  |  |  |  |  |
|  |  |  |  |  |  |
| **ITL-001/13-BS** | **OL840544** | BRESCIA | 2013 | Swine | Ag Pan + Seq |
| **macaque/64771/ITL/2012** | **OL963559** | GROSSETO | 2013 | Porcupine | Ag Pan + Seq |
| **porcupine/80843/ITL/2012** | **OL963560** | GROSSETO | 2013 | Macaque | Ag Pan + Seq |
| **ITL-004/13-PV** | **OL840543** | PAVIA | 2013 | Swine | Ag Pan + Seq |
| **ITL-007/13-MN** | **OL840542** | MANTOVA | 2013 | Swine | Ag Pan + Seq |
| **ITL-010/13-BS** | **OL840541** | BRESCIA | 2013 | Swine | Ag Pan + Seq |
| **ITL-013/13-VR **** | **OL840540** | VERONA | 2013 | Swine | Ag Pan + Seq |
| **ITL-015/13-CR *** | **OL840539** | CREMONA | 2013 | Swine | Ag Pan + Seq |
| **ITL-016/13-VR** | **OL840538** | VERONA | 2013 | Primate | Ag Pan + Seq |
| **ITL-019/13-BS** | **OL840537** | BRESCIA | 2013 | Swine | Ag Pan + Seq |
| **ITL-020/13-BS** | **OL840536** | BRESCIA | 2013 | Swine | Ag Pan + Seq |
| **ITL-021/13-BS** | **OL840535** | BRESCIA | 2013 | Swine | Ag Pan + Seq |
| **ITL-024/13-BS** | **OL840534** | BRESCIA | 2013 | Swine | Ag Pan + Seq |
| **ITL-025/13-VR** | **OL840533** | VERONA | 2013 | Primate | Ag Pan + Seq |
| **ITL-029/13-BS** | **OL840532** | BRESCIA | 2013 | Swine | Ag Pan + Seq |
| **ITL-031/13-BS** | **OL840531** | BRESCIA | 2013 | Swine | Ag Pan + Seq |
| **ITL-034/13-BS** | **OL840530** | BRESCIA | 2013 | Swine | Ag Pan + Seq |
| **ITL-035/13-PC** | **OL840529** | PIACENZA | 2013 | Swine | Ag Pan + Seq |
| **ITL-038/13-MN** | **OL840528** | MANTOVA | 2013 | Swine | Ag Pan + Seq |
| **ITL-041/13-VR** | **OL840527** | VERONA | 2013 | Primate | Ag Pan + Seq |
| **ITL-022/14-VR **** | **OL840526** | VERONA | 2014 | Swine | Ag Pan + Seq |
| **ITL-028/14-MN** | **OL840525** | MANTOVA | 2014 | Swine | Ag Pan + Seq |
| **ITL-037/14-BS ***** | **OL840524** | BRESCIA | 2014 | Swine | Ag Pan + Seq |
| **ITL-046/14-TV** | **OL840523** | PORDENONE | 2014 | Swine | Ag Pan + Seq |
| **ITL-047/14-BG** | **OL840522** | BERGAMO | 2014 | Swine | Ag Pan + Seq |
| **ITL-060/14-CR *** | **OL840521** | CREMONA | 2014 | Swine | Ag Pan + Seq |
| **ITL-065/14-CR** | **OL840520** | CREMONA | 2014 | Swine | Ag Pan + Seq |
| **ITL-076/14-PV** | **OL840519** | PAVIA | 2014 | Swine | Ag Pan + Seq |
| **ITL-082/14-MN** | **OL840518** | MANTOVA | 2014 | Swine | Ag Pan + Seq |
| **ITL-085/14-BS** | **OL840517** | BRESCIA | 2014 | Swine | Ag Pan + Seq |
| **ITL-002/15-CR** | **OL840516** | CREMONA | 2015 | Swine | Ag Pan + Seq |
| **ITL-003/15-PR** | **OL840515** | PARMA | 2015 | Swine | Ag Pan + Seq |
| **ITL-005/15-BS** | **OL840514** | BRESCIA | 2015 | Swine | Ag Pan + Seq |
| **ITL-006/15-BS** | **OL840513** | BRESCIA | 2015 | Swine | Ag Pan + Seq |
| **ITL-007/15-MO** | **OL840512** | MODENA | 2015 | Swine | Ag Pan + Seq |
| **ITL-010/15-PC** | **OL840511** | PIACENZA | 2015 | Swine | Ag Pan + Seq |
| **ITL-012/15-BS** | **OL840510** | BRESCIA | 2015 | Swine | Ag Pan + Seq |
| **ITL-015/15-BS** | **OL840509** | BRESCIA | 2015 | Swine | Ag Pan + Seq |
| **ITL-016/15-VR** | **OL840508** | VERONA | 2015 | Primate | Ag Pan + Seq |
| **ITL-026/15-PG** | **OL840507** | PERUGIA | 2015 | Swine | Ag Pan + Seq |
| **ITL-031/15-BS** | **OL840506** | BRESCIA | 2015 | Swine | Ag Pan + Seq |
| **ITL-032/15-PG** | **OL840505** | PERUGIA | 2015 | Swine | Ag Pan + Seq |
| **ITL-035/15-PC** | **OL840504** | PIACENZA | 2015 | Swine | Ag Pan + Seq |
| **ITL-042/15-BS** | **OL840503** | BRESCIA | 2015 | Swine | Ag Pan + Seq |
| **ITL-005/16-PV** | **OL840502** | PAVIA | 2016 | Swine | Ag Pan + Seq |
| **ITL-006/16-BS** | **OL840501** | BRESCIA | 2016 | Swine | Ag Pan + Seq |
| **ITL-008/16-BG** | **OL840500** | BERGAMO | 2016 | Swine | Ag Pan + Seq |
| **ITL-015/16-CR** | **OL840499** | CREMONA | 2016 | Swine | Ag Pan + Seq |
| **ITL-021/16-CN** | **OL840498** | CUNEO | 2016 | Swine | Ag Pan + Seq |
| **ITL-025/16-BS ***** | **OL840497** | BRESCIA | 2016 | Swine | Ag Pan + Seq |
| **ITL-028/16-MO** | **OL840496** | MODENA | 2016 | Swine | Ag Pan + Seq |
| **ITL-034/16-CR** | **OL840495** | CREMONA | 2016 | Swine | Ag Pan + Seq |
| **ITL-035/16-MO** | **OL840494** | MODENA | 2016 | Swine | Ag Pan + Seq |
| **ITL-036/16-PG** | **OL840493** | PERUGIA | 2016 | Swine | Ag Pan + Seq |
| **ITL-041/16-BG** | **OL840492** | BERGAMO | 2016 | Swine | Ag Pan + Seq |
| **ITL-052/16-BS** | **OL840491** | BRESCIA | 2016 | Swine | Ag Pan + Seq |
| **ITL-064/16-BG** | **OL840490** | BERGAMO | 2016 | Swine | Ag Pan + Seq |
| **ITL-079/16-RE** | **OL840489** | REGGIO EMILIA | 2016 | Swine | Ag Pan + Seq |
| **ITL-083/16-MO** | **OL840488** | MODENA | 2016 | Swine | Ag Pan + Seq |
| **ITL-090/16-LO** | **OL840487** | LODI | 2016 | Swine | Ag Pan + Seq |
| **ITL-099/16-BG** | **OL840486** | BERGAMO | 2016 | Swine | Ag Pan + Seq |
| **ITL-106/16-VR **** | **OL840485** | VERONA | 2016 | Swine | Ag Pan + Seq |
| **ITL-108/16-RA** | **OL840484** | RAVENNA | 2016 | Swine | Ag Pan + Seq |
| **ITL-007/18-RE** | **OL840483** | REGGIO EMILIA | 2018 | Swine | Ag Pan + Seq |
| **ITL-022/18-RE** | **OL840482** | REGGIO EMILIA | 2018 | Swine | Ag Pan + Seq |
| **ITL-012/19-RE** | **OL840481** | REGGIO EMILIA | 2019 | Swine | Ag Pan + Seq |
| **ITL-024/19-PG** | **OL840480** | PERUGIA | 2019 | Swine | Ag Pan + Seq |
| **ITL-032/19-RE** | **OL840479** | REGGIO EMILIA | 2019 | Swine | Ag Pan + Seq |
| **ITL-035/19-BS** | **OL840478** | BRESCIA | 2019 | Swine | Seq |
| **ITL-036-19-BS** | **OL840477** | BRESCIA | 2019 | Swine | Seq |
| **ITL-038/19-BS** | **OL840476** | BRESCIA | 2019 | Swine | Seq |
| **ITL-039/19-BS** | **OL840475** | BRESCIA | 2019 | Swine | Seq |

**Supplementary Table S1. Recent EMCV strains isolated and involved in the analysis.** This table shows the list of Italian virus isolates analysed in the work. It includes information about designation, accession number, Italian province of origin, year of sampling, hosts’ specie and analysis carried out. Asterisks sign consecutive samples from the same unique pig farm: * farm located in province of Cremona (Lombardy); ** farm located in province of Verona (Veneto); *** farm in province of Brescia (Lombardy).
