# Supplementary material for: A recent view about encephalomyocarditis virus circulating in compartmentalised animal population in Northern Italy
Source: Sci Rep. 2023 Jan 11;13:592. doi: 10.1038/s41598-023-27828-5 (PMC9834260; doi:10.1038/s41598-023-27828-5)
Supplement: Supplementary file 2 — Supplementary Information 2. [file 41598_2023_27828_MOESM2_ESM.docx]

|  |  |  |  |
| --- | --- | --- | --- |
| **Virus designation** | **Gene Acc. No.** | **Geographic origin** | **Species** |
|  |  |  |  |
|  |  |  |  |
| pig/HNJZ1201 | KF836386 | China | Swine |
| mouse/JZ1203 | KF836388 | China | Mouse |
| domasticatedboar/JZ1202 | KF836387 | China | Domesticated boar |
| Ruckert | M81861 | USA | HeLa |
| pEC9 | DQ288856 | USA | Mouse |
| pig/GXLC | FJ897755 | China | Swine |
| aardvark/HNXX13 | KF771002 | China | Aardvark |
| pig/YY13 | KF836390 | China | Swine |
| tiger/FJ13 | KF293299 | China | Tiger |
| pig/JX | KF598863 | China | Swine |
| pig/NJ08 | HM641897 | China | Swine |
| pig/BD2 | KF709977 | China | Swine |
| pig/BJC3 | DQ464062 | China | Swine |
| chimpanzee/ATCC VR-129B | KM269482 | USA | Chimpanzee |
| pig/BEL-2887A/91 | AF356822 | Belgium | Swine |
| pig/K3 | EU780148 | Rep. of Korea | Swine |
| pig/K11 | EU780149 | Rep. of Korea | Swine |
| pig/CBNU | DQ517424 | Rep. of Korea | Swine |
| pig/PV21 | X74312 | Panama | Swine |
| GX0601 | FJ604852 | China | Swine |
| pig/GS01 | KJ524643 | China | Swine |
| GX0602 | FJ604853 | China | Swine |
| pig/HB1 | DQ464063 | China | Swine |
| pig/YM13 | KF836389 | China | Swine |
| pig/HB10 | JQ864080 | China | Swine |
| pig/1029 | Y15448 | Russia | Swine |
| pig/Omsk-93 | Y15445 | Russia | Swine |
| pig/SAR/1/79 | JN800421 | Rep. of South Africa | Swine |
| pig/GRE-424/90 | AJ617362 | Greece | Swine |
| pig/EMCV-30/87 | AY296731 | USA | Swine |
| pig/NVSL-FL | X67502 | USA | Swine |
| pig/B | M22457 | Panama | Swine |
| pig/D | M22458 | Panama | Swine |
| pig/PV2 | X87335 | Panama | Swine |
| hamster/MM/usa/1942 | KP892662 | USA | Hamster |
| pig/ITL-001/96 | AJ617357 | Italy | Swine |
| pig/ITL-136/86 | AJ617358 | Italy | Swine |
| pig/CYP-108/95 | AJ617359 | Cyprus | Swine |
| pig/BEL-279/95 | AJ617361 | Belgium | Swine |
| human/Peru-2004/10855 | EU979548 | Peru | Human |
| human/Peru-2004/10854 | EU979545 | Peru | Human |
| pig/BEL-440/95 | AJ617360 | Belgium | Swine |
| rat/BEL-1086C | DQ835185 | Belgium | Rat |
| africanelephant/KNP/17/94 | JN800422 | Rep. of South Africa | African elephant |
| africanelephant/KNP/19/94 | JN800423 | Rep. of South Africa | African elephant |
| Mengo UR22-M | L22089 | Uganda | Macaque |
| baboon/3761IMP-RUS-2007 | KX231802 | Russia | Baboon |

**Supplementary Table S2. EMCV strains involved in the analysis originated from public databases.** The table displays the list of EMCV strains collected in various countries and sequenced. Only strains with at least 1D sequenced was considered. The table includes information related to virus designation, gene accession number, country of sampling and host specie.
